# Beyond Conventional Sleep Parameters: Circadian Rhythm Disruption in Adolescents with Juvenile Myoclonic Epilepsy: An Actigraphy Study

**DOI:** 10.3390/jcm15135091

**Published:** 2026-06-30

**Authors:** Ozgun Yetkin, Malgorzata Jaczak-Gozdziak, Betul Baykan, Marcin Zarowski

**Affiliations:** 1 Department of Developmental Neurology, Poznan University of Medical Sciences, 60-355 Poznan, Poland; g.jaczak@gmail.com (M.J.-G.); zarowski@ump.edu.pl (M.Z.); 2 Doctoral School, Poznan University of Medical Sciences, 60-812 Poznan, Poland; 3 Department of Neurology and Clinical Neurophysiology, EMAR Medical Center, 34367 Istanbul, Türkiye; betulbaykan@yahoo.com

**Keywords:** juvenile myoclonic epilepsy, actigraphy, circadian rhythm, chronotype, non-parametric circadian rhythm analysis, adolescents

## Abstract

**Background/Objectives:** Juvenile myoclonic epilepsy (JME) is characterized by seizures clustering in the early morning hours, reflecting circadian modulation of cortical excitability. To the best of our knowledge, circadian rest–activity rhythms have not previously been objectively characterized in adolescents with JME, a gap that may be relevant to understanding seizure timing in this population. This study aimed to characterize circadian rest–activity rhythms and sleep parameters in adolescents with JME compared to healthy controls (HCs) and to explore whether chronotype relates to the strength of circadian rhythm. **Methods:** This pilot case–control study analyzed data from 11 adolescents with JME and 10 age- and sex-matched HCs who underwent 14-day continuous wrist actigraphy. Non-parametric circadian rhythm analysis (NPCRA) and cosinor analysis were conducted, and conventional sleep–wake parameters and night-to-night variability were derived. Subjective data from the Morningness–Eveningness Questionnaire (MEQ), the Pittsburgh Sleep Quality Index, a modified Epworth Sleepiness Scale, and the Pediatric Sleep Questionnaire were included. Between-group comparisons used the Mann–Whitney U test with an effect size of r. **Results:** Patients showed significantly lower interdaily stability (*p* = 0.002, r = 0.692), higher intradaily variability (*p* = 0.022, r = 0.500), reduced peak daytime activity (*p* = 0.002, r = 0.672), and attenuated cosinor (*p* = 0.013, r = 0.544) and mesor (*p* = 0.001, r = 0.754) amplitude, all with large effect sizes. Night-to-night variability was significantly greater for sleep efficiency (*p* = 0.010, r = 0.561), the fragmentation index (*p* = 0.003, r = 0.653), and mean sleep-bout duration (*p* = 0.020, r = 0.509). The MEQ score correlated positively with cosinor amplitude (rho = 0.679, *p* = 0.022) and peak daytime activity (rho = 0.615, *p* = 0.044). No significant group differences were found in conventional sleep–wake parameters. Given the exploratory nature of this pilot study, no correction for multiple comparisons was applied, and these findings should be considered hypothesis-generating. **Conclusions:** This pilot study provides preliminary evidence of circadian rest–activity rhythm disruption in a small and clinically heterogeneous adolescent cohort, with large effect sizes in JME, despite preserved conventional sleep parameters, underscoring the potential value of NPCRA-based actigraphy monitoring in this population and the need for replication in larger cohorts.

## 1. Introduction

Juvenile myoclonic epilepsy (JME) is the most common adolescent or adult-onset idiopathic generalized epilepsy syndrome, characterized by myoclonic jerks upon awakening and generalized tonic–clonic seizures, with sleep deprivation as the most consistently reported seizure precipitant [1,2,3,4]. The characteristic clustering of seizures in the early morning hours upon awakening directly implicates circadian modulation of cortical excitability [5,6]. Several questionnaire-based studies have suggested an evening chronotype tendency in JME, though findings are inconsistent; objective assessment of the circadian phase using dim-light melatonin onset has not confirmed a definitive evening orientation, even in idiopathic generalized epilepsy [5,7]. Crucially, to the best of our knowledge, objective characterization of circadian rest–activity rhythms in adolescents with JME has not previously been performed.

Investigating these parameters in adolescents with JME presents a unique challenge, as adolescence itself imposes a biologically driven circadian delay, characterized by delayed melatonin secretion and slower accumulation of homeostatic sleep pressure, which peaks around age 20. For an adolescent with JME, early school start times impose chronic sleep deprivation at the developmental stage when the morning window is most dangerous, a clinically significant compounding effect that remains objectively uncharacterized. [8,9,10,11].

Actigraphy enables longitudinal, non-invasive ambulatory monitoring of rest–activity cycles [12]. Non-Parametric Circadian Rhythm Analysis (NPCRA) captures multidimensional circadian features without assuming a sinusoidal rhythm shape and has demonstrated superior sensitivity in clinical populations [13,14]. To date, only two actigraphy-based NPCRA studies have examined epilepsy—neither specifically in JME no in an adolescent population [13,15].

The present study aimed to characterize circadian rest–activity rhythms and sleep parameters in adolescents with JME compared with healthy controls (HCs) and to explore whether chronotype relates to the strength of objective circadian rhythms.

## 2. Materials and Methods

### 2.1. Patient Selection

This retrospective, observational, case–control study was conducted between 2024 and 2026 at the Department of Developmental Neurology, Poznan University of Medical Sciences, Poland, using medical records of adolescents with JME who had previously undergone actigraphic monitoring. Diagnosis had been established based on the typical age of onset, seizure semiology, electroencephalography (EEG) pattern, and normal neurodevelopment according to the ILAE classification [2,16].

Inclusion criteria for the JME group extracted from the databases were: (i) confirmed diagnosis of JME, (ii) age of 12–18 years, (iii) seizure freedom in the month preceding the tracking period, (iv) seizure freedom during the actigraphic monitoring period as confirmed by the archived seizure diary, and (v) absence of comorbid neurological and psychiatric disorders. Exclusion criteria for all participants included: shift work or irregular schedules; transmeridian travel in the preceding month; a known sleep disorder as defined by the International Classification of Sleep Disorders, Third Edition (ICSD-3) [17]; obstructive sleep apnea; use of hypnotics, melatonin, or medications known to affect melatonin secretion; and mood disorders. Major depressive disorder had been screened using the Beck Depression Inventory for Youth (BDI-Y II) per DSM-5 criteria [18]. Controls had undergone neurological screening and a sleep medicine interview to exclude sleep–wake disturbances.

Seizure freedom had been ascertained through patient- and caregiver-reported seizure diaries; in the absence of continuous EEG monitoring during the actigraphic recording period, subclinical or electrographic-only seizure activity could not be excluded (see Section 4.6).

All participants had records indicating they had completed a comprehensive sleep medicine interview and clinical examination. The reviewed clinical protocols consisted of one night of simultaneous polysomnography and actigraphy in a supervised clinical setting, followed by 13 consecutive nights of home actigraphy, yielding 14 days of continuous recording. A 14-day recording period was originally selected to ensure reliable estimates of interdaily stability and cosinor acrophase, both of which require extended recording for stable estimates [14], and to capture weekday–weekend variability in sleep timing, including social jetlag, across two full weekly cycles. The polysomnography night served to validate actigraphy-derived sleep–wake estimates against the gold-standard recording and to screen for occult sleep disorders not identified by clinical interview; no participants were excluded based on polysomnographic findings. Detailed polysomnographic findings, including sleep macrostructure, will be reported separately.

The study was approved by the Bioethics Committee of Poznan University of Medical Sciences (13 January 2023) and conducted in accordance with the Declaration of Helsinki and the STROBE statement [19].

### 2.2. Actigraphy Recording

Activity and ambient light data had been continuously recorded using the MotionWatch 8 actigraph (CamNTech Ltd., Cambridge, UK), a wrist-worn triaxial accelerometer validated for sleep–wake estimation in clinical and research settings [12,20]. As our sleep laboratory’s standard, the device was worn on the non-dominant wrist for 14 days, sampling in 30 s epochs. During the recording period, participants used the event-marker button at bedtime (lights out), at wake time, and before and after any daytime nap, following standard clinical instructions to maintain their daily routines and avoid adhering to specific schedules. All participants had completed a sleep diary just before going to bed each night and upon waking in the morning, recording bedtime, wake time, and the exact times of any nocturnal awakenings; patients additionally completed an epilepsy seizure log [21].

Actigraphic data were analyzed using MotionWare software (version 1.4.25; CamNTech Ltd.) with the validated medium-sensitivity threshold [20]. Each recording was visually inspected and edited by a trained investigator (O.Y.), with rest intervals verified against event markers, sleep diary entries, and light sensor data. Device removal periods were excluded per Society of Behavioral Sleep Medicine guidelines [20].

### 2.3. Actigraphic Parameters

#### 2.3.1. Sleep–Wake Parameters

Sleep–wake parameters extracted from each nightly recording included: time in bed, actual sleep time, actual wake time, sleep efficiency (defined as the ratio of AST to time in bed expressed as a percentage), sleep latency, total activity score, fragmentation index (a composite measure of sleep continuity calculated as the sum of the percentage of time spent moving and the percentage of very brief immobile episodes (Mobile Time/Immobile Bouts ≤ 1 min), with higher values indicating more disrupted, fragmented sleep), mean sleep-bout duration (the average length of continuous, uninterrupted sleep episodes occurring during the assumed sleep period), and wake-bout frequency (total number of discrete wake episodes occurring within the assumed sleep period).

The central phase measure (CPM), defined as the midpoint between sleep onset and wake time expressed in minutes past midnight, served as the primary actigraphic/behavioral proxy of circadian phase in this study. A later CPM reflects a later sleep–wake timing pattern, consistent with an evening chronotype, while an earlier CPM reflects a more morning-oriented sleep–wake pattern [11,13].

For each participant, nightly values for each sleep parameter were averaged across the 14 nights to produce a single representative value per variable. Intraindividual night-to-night variability was quantified as the standard deviation (SD) across nights. Social jetlag was calculated as the absolute difference between weekend and weekday sleep midpoints.

#### 2.3.2. Non-Parametric Circadian Rhythm Analysis

NPCRA parameters included interdaily stability (day-to-day rhythm consistency, with low interdaily stability reflecting day-to-day irregularity), intradaily variability (within-day rest–activity rhythm fragmentation, with higher values indicating a more disrupted, less consolidated rhythm), and least active 5 h window (L5; nocturnal rest level) and most active 10 h window (M10; peak daytime activity, lower M10 values indicate reduced daytime activity), along with their onset times, and relative amplitude (RA; contrast between active and rest phases, where higher values indicate robust rest–activity rhythm and lower values reflect a blunted circadian contrast) [13,22].

#### 2.3.3. Cosinor Analysis

Complementary 24 h cosinor analysis yielded the fitted cosine amplitude (overall rhythm strength), mesor (rhythm-adjusted mean activity level), and acrophase (timing of peak activity), along with daytime and nighttime average activity and the day/night ratio [22]. Daytime and nighttime average activity and the day/night activity ratio were also calculated from the 24 h activity profile.

### 2.4. Questionnaires

Sleep questionnaires were administered to the JME group at enrolment to characterize subjective sleep and chronotype profiles and to explore within-group correlates of objective actigraphic findings. Chronotype was assessed with the Morningness–Eveningness Questionnaire (MEQ; scores of 16–86, with higher scores indicating greater morningness) [23,24]. Subjective sleep quality was assessed using the Pittsburgh Sleep Quality Index (PSQI; scores of 0–21, with scores >5 indicating poor sleep quality)—specifically, the Polish-language version (PSQI-PL) [25,26,27]. Daytime sleepiness was evaluated using a 9-item version of the Epworth Sleepiness Scale (ESS) modified for children and adolescents, adapted from the original scale [28]. Sleep-related symptom burden was assessed with the Pediatric Sleep Questionnaire (PSQ) [29]. Polish-validated versions of the MEQ [24] and PSQI [25,26,27] were used; the ESS and PSQ were administered as departmental translations of the original validated instruments.

### 2.5. Statistical Analysis

Statistical analyses were performed using R (version 4.5.2; R Core Team, Vienna, Austria). Claude AI (Anthropic, Claude Sonnet) was used to assist in the generation and debugging of R code for these analyses; all outputs were reviewed and validated by the authors. Continuous variables are expressed as medians and interquartile ranges (IQRs), and categorical variables are expressed as frequencies and percentages. Given the small sample size and the predominantly non-normal distributions of the actigraphic variables, as confirmed by the Shapiro–Wilk test, non-parametric tests were used throughout. Between-group comparisons were performed using the Mann–Whitney U test for continuous variables and Fisher’s exact test for categorical variables. The significance level was set at *p* < 0.05 (two-tailed). Effect sizes were calculated as r = Z/√N and interpreted according to Cohen’s conventions (small: r = 0.1; medium: r = 0.3; large: r = 0.5) [30,31,32]. Within-group correlations in the JME group were assessed using Spearman’s rank correlation coefficient (ρ). Given the rarity of the study population and the single-center design, this study was conceived as a pilot investigation; sample-size calculations for future confirmatory studies will be informed by the effect sizes reported here. No corrections for multiple comparisons were applied, given the exploratory nature of this pilot study; findings should therefore be interpreted with appropriate caution.

## 3. Results

### 3.1. Participants and Clinical Characteristics

A total of 22 medical records were initially screened, comprising 12 adolescents with JME and 10 HCs. One JME patient’s record was excluded because the historical monitoring period was incomplete due to a documented allergic skin reaction to the actigraphy device, and the patient was unable to complete it, resulting in a final sample of 21 participants (11 JME subjects and 10 HCs). The groups were well matched for age (both groups: median, 16 years; U = 65.5; *p* = 0.416) and sex distribution (HCs, 7F/3M; JME, 6F/5M; Fisher’s exact *p* = 0.659). Clinical characteristics of the JME group are summarized in Table 1. Six patients (54.5%) had achieved seizure remission under treatment, while five (45.5%) had ongoing seizures despite treatment. All patients were on antiepileptic medication (ASM) at the time of their initial actigraphic monitoring.

### 3.2. Conventional Sleep–Wake Parameters

No statistically significant differences were observed between groups in any of the conventional actigraphy-derived sleep–wake parameters. Time in bed, actual sleep time, actual wake time, sleep efficiency, sleep latency, fragmentation index, total activity score, CPM, mean sleep-bout duration, and wake-bout frequency were all comparable between groups (all *p* > 0.05). Nocturnal ambient light levels were similarly comparable (HCs: median, 1.52 lux; IQR, 1.51; JME: median, 0.95 lux; IQR, 1.47; U = 58.0; *p* = 0.863; r = 0.038). Full descriptive statistics are presented in Table 2.

### 3.3. Sleep Timing and Social Jetlag

Sleep timing and social jetlag data are presented in Table 2. On weekdays, median sleep onset was 23:12 in the JME group and 22:58 in HCs, with comparable wake times (07:13 and 07:23, respectively). On weekends, HCs showed delays in both sleep onset (00:31) and wake time (09:26). In contrast, the JME group showed a comparatively smaller delay (sleep onset, 23:56; wake time, 08:34). Social jetlag was numerically lower in the JME group (median, 54.7 min; IQR, 49.6) than in HCs (median, 102.5 min; IQR, 73.1). However, this difference did not reach statistical significance (U = 80.0; *p* = 0.085; r = 0.376).

### 3.4. Circadian Rhythm Parameters

NPCRA and cosinor parameters are summarized in Table 3 and Table 4.

Significant group differences were observed in six of the eleven examined circadian variables. Interdaily stability was significantly lower in the JME group (median, 0.34; IQR, 0.09) compared to HCs (median, 0.48; IQR, 0.15; U = 98; *p* = 0.002; r = 0.692). Intradaily variability was significantly higher in the JME group (median, 0.99; IQR, 0.33) than in HCs (median, 0.67; IQR, 0.18; U = 22; *p* = 0.022; r = 0.500). M10 average activity was significantly lower in the JME group (median, 13,120 activity counts; IQR 3044) compared to HCs (median, 19,249 activity counts; IQR, 8458; U = 97; *p* = 0.002; r = 0.672). L5 and relative amplitude and M10 and L5 onset times did not differ significantly between groups (all *p* > 0.05).

**Figure 1:**
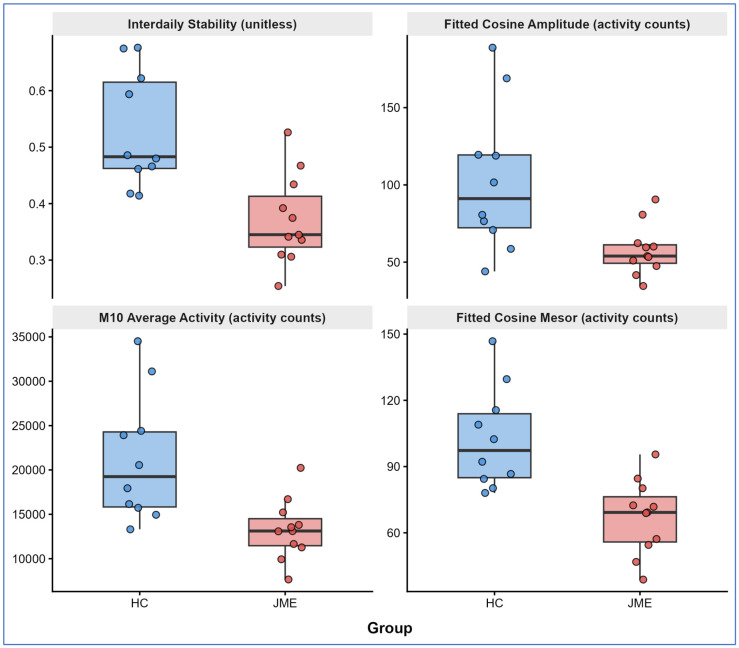
Individual-participant values for selected non-parametric circadian rhythm analysis (NPCRA) and cosinor parameters by group. Boxplots show medians and interquartile ranges; points represent individual participants. Interdaily stability is unitless (range 0–1); M10 average activity, fitted cosine amplitude, and fitted cosine mesor are expressed as activity counts. Abbreviations: HC, healthy control; JME: juvenile myoclonic epilepsy.

Cosinor analysis revealed significantly lower fitted cosine amplitudes (HCs: median, 91.1; IQR, 47.2; JME: median, 53.9; IQR 12.0; U = 90; *p* = 0.013; r = 0.544), cosine mesors (HCs: median, 97.3; IQR, 29.0; JME: median, 69.2; IQR 20.5; U = 101; *p* = 0.001; r = 0.754), and daytime average activity (HCs: median, 130; IQR, 44.4; JME: median, 96.5; IQR, 27.9; U = 100; *p* = 0.001; r = 0.733) in the JME group. Acrophase, nighttime average activity, and the day/night activity ratio did not differ significantly between groups (*p* = 0.809, 0.152, and 0.918, respectively). All significant differences demonstrated large effect sizes (r = 0.500–0.754) (Figure 1).

### 3.5. Night-to-Night Sleep Variability

Night-to-night variability data are presented in Table 4. The standard deviation of sleep efficiency was significantly higher in the JME group (median, 6.04%; IQR, 7.09) than in HCs (median, 3.13%; IQR, 1.30; U = 19; *p* = 0.010; r = 0.561). Night-to-night variability in the fragmentation index was significantly higher in the JME group (median, 10.4; IQR, 3.36) than HCs (median, 5.57; IQR, 2.43; U = 14; *p* = 0.003; r = 0.653). Variability in mean sleep-bout duration was also significantly higher in the JME group (median, 378 min; IQR, 449) relative to HCs (median, 178 min; IQR, 132; U = 22; *p* = 0.020; r = 0.509). Variability in the CPM did not differ significantly between groups (HCs: median, 35.1 min; IQR, 46.9; JME: median, 51.6 min; IQR, 26.4; U = 34; *p* = 0.152; r = 0.313).

### 3.6. Within-Group Correlations in JME

Spearman correlation analyses revealed significant positive correlations between MEQ score and fitted cosine amplitude (rho = 0.679, *p* = 0.022) and between MEQ score and average M10 activity (rho = 0.615, *p* = 0.044). No significant correlations were observed between disease duration and IV or between ESS score and CPM variability (Table 5).

### 3.7. Questionnaire Results

Questionnaire data were available for all 11 JME patients. The MEQ indicated a predominantly intermediate chronotype profile (9 intermediate, 1 moderate evening, and 1 moderate morning; median score, 52; IQR, 11.5). The median PSQI score was 6 (IQR, 3), with most patients exceeding the clinical cutoff of 5. The median Carskadon-modified ESS score was 3 (IQR, 8.5). The PSQ yielded a median total symptom score of 10 (IQR, 2.5) out of a possible 56.

### 3.8. Seizure-Control Status and ASM Class

Given the clinical heterogeneity of the JME group, key sleep–wake and circadian parameters were additionally descriptively examined by seizure-control status and ASM class (Table 6). No formal statistical comparisons were performed, given the small subgroup sizes (ASM subgroups ranging from *n* = 1 to *n* = 6). Seizure-control status and ASM class showed substantial overlap in this cohort, as five of the six remission patients were on levetiracetam monotherapy, limiting the ability to disentangle medication-specific from seizure-control-specific associations.

## 4. Discussion

### 4.1. Circadian Rhythm Disruption in JME

This pilot study provides the first objective characterization of circadian rest–activity rhythms in adolescents with JME, with findings suggesting significantly lower interdaily stability, higher intradaily variability, reduced M10, and attenuated cosinor amplitude and mesor. These findings replicate and extend those of Liguori et al. in a mixed adult epilepsy cohort [13] and are consistent with a recent large-scale National Health and Nutrition Examination Survey (NHANES)-based study that confirmed lower interdaily stability and M10 in patients with epilepsy compared to the general population [15]. The present cohort suggests that this disruption is detectable specifically in adolescents with JME, a population in which epilepsy-related circadian dysregulation may compound the physiologically delayed circadian phase that characterizes normal adolescent development [10,11]. The attenuated cosinor amplitude and mesor further suggest a globally suppressed activity profile in JME, beyond rhythm fragmentation alone.

### 4.2. Dissociation Between Circadian and Conventional Sleep Parameters

In contrast to the large circadian rhythm disparities, no significant differences were observed in conventional sleep–wake metrics, including sleep efficiency, sleep latency, time in bed, and fragmentation index. This dissociation suggests that circadian dysregulation in JME may manifest primarily at the level of rhythm quality and regularity rather than gross changes in mean sleep parameters, a pattern detectable only through NPCRA, underscoring its added value over conventional actigraphy metrics in this population [13].

The absence of objective differences in mean sleep parameters may partly reflect the clinical heterogeneity of the sample. The JME group comprised patients with varying seizure control status (remission in six and ongoing seizures in five) and different ASM regimens (levetiracetam, valproate, lamotrigine, and polytherapy). These factors may have exerted differential effects on sleep architecture and circadian rhythm parameters. To investigate this, we descriptively compared key parameters by seizure-control status and ASM class (Table 6). However, the significant overlap between these groupings in our small cohort limits the conclusions that can be drawn from it.

Valproate has been shown to reduce REM sleep, increase N1 sleep, and increase nocturnal awakenings in patients with epilepsy [33]. At the same time, levetiracetam has been reported to consolidate sleep by increasing sleep efficiency and reducing stage shifts [34]. Lamotrigine treatment has been associated with increased REM sleep and reduced stage shifts and arousals [35]. Animal studies further suggest that valproate may lengthen the circadian rhythm [36].

Although the small subgroup sizes preclude formal statistical comparison, the descriptive data in Table 6 are broadly consistent with this literature: LEV-treated patients showed somewhat higher sleep efficiency and a lower fragmentation index than VPA-treated patients. Given the very small size of the VPA subgroup (*n* = 2) and the overlap between ASM class and seizure-control status in this cohort, this observation should be regarded as hypothesis-generating rather than confirmatory.

Therefore, the heterogeneous ASM exposure in our cohort may have introduced variability, obscuring both group-level mean differences and within-group circadian patterns. Future research in ASM-naïve patients or with controlled medication withdrawal designs will be needed to disentangle disease-related from treatment-related effects on circadian disruption.

### 4.3. Neurobiological Mechanisms and Clinical Implications

The thalamocortical network dysfunction underlying JME is also central to sleep–wake regulation and circadian rhythmicity [5]. The characteristic clustering of JME seizures in the early morning hours following sleep–wake transition directly reflects this circadian influence on cortical excitability [6]. This transition represents a particularly vulnerable window; the abrupt shift from sleep-related inhibitory states to wakefulness temporarily destabilizes thalamocortical synchrony, lowering the seizure threshold precisely when circadian alerting signals are rising [6]. Notably, longer epileptiform discharges surge specifically at wake onset and remain frequent throughout the waking period, while shorter discharges predominate during sleep, a pattern that directly mirrors the clinical seizure distribution [37].

The reduced interdaily stability and increased intradaily variability observed in this cohort suggest that this vulnerable morning window may vary in intensity from day to day, as a poorly synchronized circadian rhythm may generate inconsistent sleep–wake transitions and fluctuating levels of thalamocortical excitability [6]. In this framework, circadian dysregulation in JME may actively modulate day-to-day seizure risk expression within the already established morning vulnerability window [38]. Therefore, identifying adolescents with JME who show the greatest circadian fragmentation may guide targeted sleep hygiene interventions and chronobiologically optimized ASM timing [39].

### 4.4. Night-to-Night Sleep Variability

A novel finding of this study is the significantly greater night-to-night variability in sleep efficiency, fragmentation index, and mean sleep-bout duration in the JME group compared to HCs, as quantified by 14-night intraindividual standard deviations. This sleep instability represents a dimension of sleep disruption distinct from mean parameter differences and has not been previously reported in JME. It is clinically relevant, as a reduction in sleep efficiency on a given night has been associated with an increased risk of seizures the following day, with a 1.25-fold increase in seizure odds per standard-deviation decrease in sleep efficiency [38]. Therefore, greater night-to-night sleep instability in JME may represent not only a marker of dysregulated sleep–wake regulation but also a potential contributor to the unpredictable day-to-day seizure pattern characteristic of this syndrome [5].

### 4.5. Chronotype and Circadian Rhythm Strength

Our cohort had a predominantly intermediate—rather than strongly evening— chronotype, consistent with the findings of Manni et al., who also reported that neither subjective MEQ scores nor objective circadian phase markers indicated a definite evening orientation in patients with idiopathic generalized epilepsy [7]. This trend may also represent the confounding influence of the biological circadian delay inherent in adolescence, which may disguise a disease-specific evening chronotype preference in this age range [10,11].

In the JME group, the MEQ score was strongly associated with both the fitted cosine amplitude and the average M10 activity, suggesting that individuals with a more morning-oriented chronotype had stronger circadian rhythms and greater peak daily activity. A morning chronotype has been associated with greater synchronization of the internal circadian clock with the external light–dark cycle, which, in turn, promotes greater circadian amplitude and higher levels of daytime activity [11,40]. Conversely, a more evening-oriented chronotype was associated with lower circadian amplitude and daytime peak activity, consistent with the circadian misalignment framework in JME [7]. These findings add an objective dimension to the previously reported subjective evening chronotype tendency in JME [41], suggesting that chronotype has measurable consequences for circadian rhythm strength beyond subjective preference.

### 4.6. Limitations

Several limitations of this study should be acknowledged. First, the sample size is small, reflecting the challenges of recruiting a homogeneous adolescent JME cohort with strict inclusion and exclusion criteria at a single center; findings should therefore be interpreted with caution and require replication in larger, ideally multicenter cohorts. Second, given the small sample size and the exploratory nature of this pilot study, no correction for multiple comparisons was applied; consequently, borderline findings, including the higher intradaily variability in JME (*p* = 0.022) and the within-group correlations between MEQ score and both cosinor amplitude (*p* = 0.022) and average M10 activity (*p* = 0.044), should be interpreted with particular caution and regarded as hypothesis-generating rather than confirmatory.

Third, the clinical heterogeneity of the JME group with respect to seizure control status and ASM regimen may have contributed to within-group variability in circadian and sleep parameters, as discussed above. Fourth, all patients were on ASM throughout the recording period, limiting the ability to distinguish disease-related from treatment-related effects on circadian rhythm measures. Fifth, the absence of simultaneous EEG recordings means that interictal epileptiform activity cannot be excluded and may have independently affected sleep and circadian parameters. Sixth, actigraphy, while validated for sleep–wake estimation in children with epilepsy, cannot discriminate sleep stages or detect epileptiform activity and may misclassify quiet wakefulness as sleep [12]. Seventh, questionnaire data were collected only in the JME group, precluding between-group comparisons of subjective sleep quality and chronotype. Eighth, seasonal variation in ambient light exposure, a critical circadian zeitgeber, may have independently influenced NPCRA parameters. Finally, the cross-sectional design precludes causal inference regarding the relationship between circadian dysregulation and seizure susceptibility in JME.

## 5. Conclusions

This pilot study provides preliminary evidence that adolescents with JME may exhibit disruption of circadian rest–activity rhythms, characterized by reduced IS, increased IV, attenuated circadian amplitude and mesor, and reduced peak daytime activity in the absence of significant differences in conventional mean sleep parameters. Greater night-to-night sleep instability represents an additional and previously unreported dimension of sleep dysregulation in this population. A morning chronotype was associated with stronger circadian rhythm parameters, suggesting the potential clinical relevance of chronotype assessment in JME. These findings should be interpreted in the context of the study group’s small sample size and clinical heterogeneity, including variability in seizure control status and ASM regimens. If replicated in larger, more homogeneous cohorts, these results would support the integration of circadian monitoring into the clinical care of adolescents with JME and the exploration of circadian-targeted interventions to mitigate seizure risk.

## Figures and Tables

**Table 1:** Clinical characteristics of the JME group.

Variable	JME (*n* = 11)	HC (*n* = 10)	*p*
Age, median (IQR)	16 (0)	16 (1)	0.416
Sex, F/M	6/5	7/3	0.659
Age at epilepsy onset, median (IQR)	14 (1)	-	-
Time since diagnosis (months), median (IQR)	5 (15.5)	-	-
Seizure control, *n* (%)	Remission: 6 (54.5%)/ Ongoing seizures despite treatment: 5 (45.5%)	-	-
Anti-seizure medication, *n* (%)	LEV: 6 (54.5%); VPA: 2 (18.2%); LTG: 1 (9.1%); Polytherapy: 2 (18.2%)	-	-

Data are presented as median (IQR) or *n* (%). Polytherapy: two or more ASMs. Abbreviations: IQR, interquartile range; LEV, levetiracetam; VPA, valproic acid; LTG, lamotrigine; F, female; M, male; JME, juvenile myoclonic epilepsy; HC, healthy control; *n*, number.

**Table 2:** Actigraphy-derived sleep–wake parameters.

Variable	Controls (*n* = 10) Median (IQR)	JME (*n* = 11) Median (IQR)	U	*p*	r
TIB, min	525.0 (88.9)	548.0 (47.1)	50.5	0.778	0.061
AST, min	473.0 (62.1)	444.7 (52.4)	63.0	0.605	0.113
AWT, min	40.7 (27.8)	31.6 (22.1)	62.5	0.622	0.108
Sleep efficiency, %	87.6 (4.98)	85.3 (7.91)	73.0	0.223	0.266
Sleep latency, min	16.8 (18.0)	22.2 (39.3)	35.0	0.173	0.297
Fragmentation index	22.8 (13.2)	22.4 (6.16)	54.0	0.973	0.008
Total activity score (activity counts)	5307 (4426)	3959 (3403)	67.0	0.426	0.174
CPM, min past midnight	238 (108)	214 (64.2)	70.0	0.314	0.220
Mean sleep bouts, min	834 (256)	1163 (331)	31.0	0.099	0.360
Wake bouts, *n*	34.1 (19.9)	30.7 (11.1)	60.0	0.756	0.068
Average light, lux	1.52 (1.51)	0.95 (1.47)	58.0	0.863	0.038
Sleep onset—weekdays	22:58	23:12	—	—	—
Sleep onset—weekends	00:31	23:56	—	—	—
Wake time—weekdays	07:23	07:13	—	—	—
Wake time—weekends	09:26	08:34	—	—	—
Social jetlag, min	102.5 (73.1)	54.7 (49.6)	80.0	0.085	0.376

Data are presented as medians (IQR). Sleep timing data are presented as median clock times. Abbreviations: IQR, interquartile range; JME, juvenile myoclonic epilepsy; TIB, time in bed; AST, actual sleep time; AWT, actual wake time; CPM, central phase measure; U, Mann–Whitney U statistic; r, effect size.

**Table 3:** Non-Parametric Circadian Rhythm Analysis (NPCRA) parameters.

Variable	Controls (*n* = 10) Median (IQR)	JME (*n* = 11) Median (IQR)	U	*p*	r
Interdaily stability	0.48 (0.15)	0.34 (0.09)	98	**0.002**	0.692
Intradaily variability	0.67 (0.18)	0.99 (0.33)	22	**0.022**	0.500
M10 average activity	19,249 (8458)	13,120 (3044)	97	**0.002**	0.672
M10 start time	08:30	08:00	48.5	0.667	0.094
L5 average activity	792 (304)	495 (673)	65	0.512	0.143
L5 start time	01:00	00:00	74.5	0.155	0.310
Relative amplitude	0.90 (0.06)	0.91 (0.10)	67	0.418	0.177

Data are presented as medians (IQR). Start times are presented as median clock times. Abbreviations: IQR, interquartile range; JME, juvenile myoclonic epilepsy; M10, most active 10 h window; L5, least active 5 h window; U, Mann–Whitney U statistic; r, effect size.

**Table 4:** Cosinor parameters and night-to-night variability.

Variable	Controls (*n* = 10) Median (IQR)	JME (*n* = 11) Median (IQR)	U	*p*	r
**Cosinor parameters**					
Fitted cosine amplitude	91.1 (47.2)	53.9 (12.0)	90	**0.013**	0.544
Fitted cosine mesor	97.3 (29.0)	69.2 (20.5)	101	**0.001**	0.754
Fitted cosine peak (acrophase)	14:27	14:31	51.0	0.809	0.053
Daytime average activity	130 (44.4)	96.5 (27.9)	100	**0.001**	0.733
Nighttime average activity	19.09 (9.81)	12.4 (5.17)	76	0.152	0.313
Average day/night ratio	7.98 (6.39)	7.77 (3.48)	57.0	0.918	0.023
**Night-to-night variability**					
Sleep efficiency SD, %	3.13 (1.30)	6.04 (7.09)	19	**0.010**	0.561
Fragmentation index SD	5.57 (2.43)	10.4 (3.36)	14	**0.003**	0.653
Mean sleep-bout SD, min	178 (132)	378 (449)	22	**0.020**	0.509
CPM SD, min	35.1 (46.9)	51.6 (26.4)	34	0.152	0.313

Data are presented as medians (IQR). The acrophase is presented as the median clock time. SD values represent intraindividual night-to-night variability across 14 recording nights. Abbreviations: IQR, interquartile range; JME, juvenile myoclonic epilepsy; SD, standard deviation; CPM, central phase measure; U: Mann–Whitney U statistic; r, effect size.

**Table 5:** Spearman correlation analysis within the JME group.

Variable 1	Variable 2	rho	*p*
MEQ score	Fitted cosine amplitude	0.679	**0.022**
MEQ score	M10 average activity	0.615	**0.044**
Disease duration	IV	0.406	0.215
ESS score	CPM SD	0.507	0.111

Statistically significant correlations (*p* < 0.05) are shown in bold. Abbreviations: JME, juvenile myoclonic epilepsy; MEQ, Morningness–Eveningness Questionnaire; M10, most active 10 h window; IV, intradaily variability; CPM, central phase measure; SD, standard deviation; ESS, Epworth Sleepiness Scale; rho, Spearman’s correlation coefficient.

**Table 6:** Descriptive subgroup analysis by seizure-control status and ASM class.

Variable	Ongoing (*n* = 5)	Remission (*n* = 6)	LEV (*n* = 6)	LTG (*n* = 1)	Polytherapy (*n* = 2)	VPA (*n* = 2)
Sleep efficiency, %	85.3 (1.9)	87.4 (8.6)	85.3 (7.5)	89.8 (n/a)	88.6 (4.9)	80.0 (5.3)
Fragmentation index	22.9 (7.9)	21.2 (2.3)	22.2 (2.4)	20.8 (n/a)	24.3 (6.9)	26.4 (4.0)
Mean sleep bout, min	843.0 (429.2)	1209.8 (252.5)	1209.8 (252.5)	915.9 (n/a)	966.2 (232.0)	912.5 (251.0)
Interdaily stability	0.3 (0)	0.4 (0.1)	0.4 (0.1)	0.5 (n/a)	0.4 (0)	0.3 (0)
Intradaily variability	1.1 (0.1)	0.8 (0.2)	0.8 (0.3)	0.8 (n/a)	1.1 (0)	1.1 (0.1)
Fitted cosine amplitude	53.9 (12.1)	57.8 (24.5)	56.4 (10.0)	80.7 (n/a)	57.0 (3.1)	41.0 (6.5)

Data are presented as medians (interquartile range) for descriptive purposes; no formal statistical comparisons were performed, given the small subgroup sizes. Seizure-control status and ASM class showed substantial overlap in this cohort (5 of 6 remission patients were on LEV monotherapy), limiting the ability to disentangle medication-specific from seizure-control-specific associations. Abbreviations: ASM, anti-seizure medication; LEV, levetiracetam; LTG, lamotrigine; VPA, valproic acid.

## Data Availability

The original contributions presented in this study are included in the article. Further inquiries can be directed to the corresponding author.

## Abbreviations

The following abbreviations are used in this manuscript:

ASM	Anti-seizure medication
BDI-Y II	Beck Depression Inventory for Youth
CPM	Central phase measure
EEG	Electroencephalography
ESS	Epworth Sleepiness Scale
HC	Healthy control
ICSD-3	International Classification of Sleep Disorders, Third Edition
IQR	Interquartile range
JME	Juvenile myoclonic epilepsy
L5	Least active 5 h window
M10	Most active 10 h window
MEQ	Morningness–Eveningness Questionnaire
NHANES	National Health and Nutrition Examination Survey
NPCRA	Non-parametric circadian rhythm analysis
PSQ	Pediatric Sleep Questionnaire
PSQI	Pittsburgh Sleep Quality Index
SD	Standard deviation
STROBE	Strengthening the Reporting of Observational Studies in Epidemiology

## References

[1] Striano P., Nobile C. (2018). The genetic basis of juvenile myoclonic epilepsy. Lancet Neurol..

[2] Hirsch E., French J., Scheffer I.E., Bogacz A., Alsaadi T., Sperling M.R., Abdulla F., Zuberi S.M., Trinka E., Specchio N. (2022). ILAE definition of the Idiopathic Generalized Epilepsy Syndromes: Position statement by the ILAE Task Force on Nosology and Definitions. Epilepsia.

[3] Stevelink R., Koeleman B.P.C., Sander J.W., Jansen F.E., Braun K.P.J. (2019). Refractory juvenile myoclonic epilepsy: A meta-analysis of prevalence and risk factors. Eur. J. Neurol..

[4] Manganotti P., Bongiovanni L.G., Fuggetta G., Zanette G., Fiaschi A. (2006). Effects of sleep deprivation on cortical excitability in patients affected by juvenile myoclonic epilepsy: A combined transcranial magnetic stimulation and EEG study. J. Neurol. Neurosurg. Psychiatry.

[5] Yetkin O., Zarowski M., Baykan B. (2024). Sleep in juvenile myoclonic epilepsy: A systematic review. Seizure.

[6] Badawy R.A., Macdonell R.A., Jackson G.D., Berkovic S.F. (2009). Why do seizures in generalized epilepsy often occur in the morning?. Neurology.

[7] Manni R., De Icco R., Cremascoli R., Ferrera G., Furia F., Zambrelli E., Canevini M.P., Terzaghi M. (2016). Circadian phase typing in idiopathic generalized epilepsy: Dim light melatonin onset and patterns of melatonin secretion-Semicurve findings in adult patients. Epilepsy Behav..

[8] Sivertsen B., Pallesen S., Stormark K.M., Bøe T., Lundervold A.J., Hysing M. (2013). Delayed sleep phase syndrome in adolescents: Prevalence and correlates in a large population based study. BMC Public Health.

[9] Gradisar M., Gardner G., Dohnt H. (2011). Recent worldwide sleep patterns and problems during adolescence: A review and meta-analysis of age, region, and sleep. Sleep Med..

[10] Hagenauer M.H., Perryman J.I., Lee T.M., Carskadon M.A. (2009). Adolescent changes in the homeostatic and circadian regulation of sleep. Dev. Neurosci..

[11] Roenneberg T., Kuehnle T., Pramstaller P.P., Ricken J., Havel M., Guth A., Merrow M. (2004). A marker for the end of adolescence. Curr. Biol..

[12] Sadaka Y., Sadeh A., Bradbury L., Massicotte C., Zak M., Go C., Shorer Z., Weiss S.K. (2014). Validation of actigraphy with continuous video-electroencephalography in children with epilepsy. Sleep Med..

[13] Liguori C., Spanetta M., Fernandes M., Izzi F., Placidi F., Mercuri N.B. (2022). More than sleep and wake disturbances: An actigraphic study showing the sleep-wake pattern dysregulation in epilepsy. Seizure.

[14] Gao C., Haghayegh S., Wagner M., Cai R., Hu K., Gao L., Li P. (2023). Approaches for Assessing Circadian Rest-Activity Patterns Using Actigraphy in Cohort and Population-Based Studies. Curr. Sleep Med. Rep..

[15] Tang T., Zhou Y., Zhai X. (2024). Circadian rhythm and epilepsy: A nationally representative cross-sectional study based on actigraphy data. Front. Neurol..

[16] Beniczky S., Trinka E., Wirrell E., Abdulla F., Al Baradie R., Alonso Vanegas M., Auvin S., Singh M.B., Blumenfeld H., Bogacz Fressola A. (2025). Updated classification of epileptic seizures: Position paper of the International League Against Epilepsy. Epilepsia.

[17] Sateia M.J. (2014). International classification of sleep disorders-third edition: Highlights and modifications. Chest.

[18] Beck J.S., Beck A.T., Jolly J.B. (2001). Manual for the Beck Youth Inventories of Emotional and Social Impairment.

[19] von Elm E., Altman D.G., Egger M., Pocock S.J., Gøtzsche P.C., Vandenbroucke J.P. (2007). The Strengthening the Reporting of Observational Studies in Epidemiology (STROBE) statement: Guidelines for reporting observational studies. Lancet.

[20] Ancoli-Israel S., Martin J.L., Blackwell T., Buenaver L., Liu L., Meltzer L.J., Sadeh A., Spira A.P., Taylor D.J. (2015). The SBSM Guide to Actigraphy Monitoring: Clinical and Research Applications. Behav. Sleep Med..

[21] Smith M.T., McCrae C.S., Cheung J., Martin J.L., Harrod C.G., Heald J.L., Carden K.A. (2018). Use of Actigraphy for the Evaluation of Sleep Disorders and Circadian Rhythm Sleep-Wake Disorders: An American Academy of Sleep Medicine Clinical Practice Guideline. J. Clin. Sleep Med..

[22] Van Someren E.J., Swaab D.F., Colenda C.C., Cohen W., McCall W.V., Rosenquist P.B. (1999). Bright light therapy: Improved sensitivity to its effects on rest-activity rhythms in Alzheimer patients by application of nonparametric methods. Chronobiol. Int..

[23] Horne J.A., Ostberg O. (1976). A self-assessment questionnaire to determine morningness-eveningness in human circadian rhythms. Int. J. Chronobiol..

[24] Jankowski K.S. (2013). Polish version of the reduced Morningness–Eveningness Questionnaire. Biol. Rhythm Res..

[25] Buysse D.J., Reynolds C.F., Monk T.H., Berman S.R., Kupfer D.J. (1989). The Pittsburgh Sleep Quality Index: A new instrument for psychiatric practice and research. Psychiatry Res..

[26] de la Vega R., Tomé-Pires C., Solé E., Racine M., Castarlenas E., Jensen M.P., Miró J. (2015). The Pittsburgh Sleep Quality Index: Validity and factor structure in young people. Psychol. Assess..

[27] Olech M., Brzezińska A., Biernacka M., Bochniarz K.T., Jurek P. (2025). Psychometryczne Właściwości i Zastosowanie Polskiej Wersji Pittsburskiego Indeksu Jakości Snu (PSQI-PL).

[28] Johns M.W. (1991). A new method for measuring daytime sleepiness: The Epworth sleepiness scale. Sleep.

[29] Chervin R.D., Hedger K., Dillon J.E., Pituch K.J. (2000). Pediatric sleep questionnaire (PSQ): Validity and reliability of scales for sleep-disordered breathing, snoring, sleepiness, and behavioral problems. Sleep Med..

[30] Gignac G.E., Szodorai E.T. (2016). Effect size guidelines for individual differences researchers. Personal. Individ. Differ..

[31] Fiel Peres F. (2026). Effect sizes for nonparametric tests. Biochem. Med..

[32] Cohen J. (1988). Statistical Power Analysis for the Behavioral Sciences.

[33] Zhang H., Li Y., Li X., Liu G., Wang B., Li C. (2014). Effect of sodium valproate on the sleep structures of epileptic patients. Exp. Ther. Med..

[34] Cicolin A., Magliola U., Giordano A., Terreni A., Bucca C., Mutani R. (2006). Effects of levetiracetam on nocturnal sleep and daytime vigilance in healthy volunteers. Epilepsia.

[35] Foldvary N., Perry M., Lee J., Dinner D., Morris H.H. (2001). The effects of lamotrigine on sleep in patients with epilepsy. Epilepsia.

[36] Næsgaard J.A.R., Gjerstad L., Heuser K., Taubøll E. (2023). Biological rhythms and epilepsy treatment. Front. Neurol..

[37] Turco F., Giorgi F.S., Maestri M., Morganti R., Benedetto A., Milano C., Pizzanelli C., Menicucci D., Gemignani A., Fornai F. (2021). Prolonged and short epileptiform discharges have an opposite relationship with the sleep–wake cycle in patients with JME: Implications for EEG recording protocols. Epilepsy Behav..

[38] Gagliano L., Ding T.Y., Toffa D.H., Beauregard L., Robert M., Lesage F., Sawan M., Nguyen D.K., Bou Assi E. (2022). Decrease in wearable-based nocturnal sleep efficiency precedes epileptic seizures. Front. Neurol..

[39] Rehim E., Li S.P., Vendrame M. (2023). Seizure control with treatment of delayed sleep-wake phase disorder in juvenile myoclonic epilepsy: A case report. Epilepsy Behav. Rep..

[40] Roenneberg T., Kuehnle T., Juda M., Kantermann T., Allebrandt K., Gordijn M., Merrow M. (2007). Epidemiology of the human circadian clock. Sleep Med. Rev..

[41] Koike C., Lima E.M., Paiva M.L., Pentagna A., Bimbatti I., Valente K.D. (2023). Sleep quality and circadian rhythm profile of persons with juvenile myoclonic epilepsy in a tertiary epilepsy center: A case-control study. Seizure.

